# Association of periodic limb movements during sleep and tinnitus in humans

**DOI:** 10.1038/s41598-020-62987-9

**Published:** 2020-04-06

**Authors:** Sheng-Wei Hwang, Yia-Chi Chu, Shang-Rung Hwang, Juen-Haur Hwang

**Affiliations:** 1 Da Yeh Junior High School, Taichung, Taiwan; 2 Taichung Girls Senior High School, Taichung, Taiwan; 3 Department of Otolaryngology-Head and Neck Surgery, Dalin Tzu Chi Hospital, Buddhist Tzu Chi Medical Foundation, Chiayi, Taiwan; 4 School of Medicine, Tzu Chi University, Hualien, Taiwan; 5 Department of Medical Research, China Medical University Hospital, China Medical University, Taichung, Taiwan

**Keywords:** Medical research, Outcomes research

## Abstract

Both of periodic limb movements during sleep (PLMS) and tinnitus were related with dopaminergic system dysfunction. However, it was still unclear whether PLMS, one kind of sleep disturbances, was associated with chronic tinnitus or not. Thus, we aimed to investigate this issue in humans. Clinical and overnight polysomnographic data of 2849 adults from a community hospital during Nov. 2011 to Jun 2017 in Taiwan was collected retrospectively. The association of PLMS and chronic tinnitus was analyzed by Student’s t-test, Pearson’s Chi-Square test, and multivariate logistic regression. The results showed that **t**he mean age was 50.6 years old (standard deviation, SD = 13.3, range = 18~91) for all subjects. There were 1886 subjects without tinnitus and 963 subjects with tinnitus in this study. The PLMS was not significantly different between subjects without tinnitus (mean = 1.0/h, SD = 3.5/h) and subjects with tinnitus mean = 1.1/h, SD = 3.4/h) by Student’s t-test. The severity of PLMS was not significantly between non-tinnitus and tinnitus subjects by Pearson’s Chi-Square test. Multivariate logistic regression also showed that PLMS was not significantly associated with tinnitus after adjusting age, sex, subjective hearing loss, Parkinson’s disease, and insomnia. In conclusion, PLMS was not associated with chronic tinnitus in humans.

## Introduction

Tinnitus is a phantom auditory perception. There were many types of tinnitus, for example auditory tinnitus, somatic tinnitus, vascular origin tinnitus, muscular origin tinnitus, etc. And, the prevalence of tinnitus depends on the type and/or definition for it. A nationwide survey of South Korea reported that the prevalence of any tinnitus was 20.7% among adults aged 20 to 98 years^1^. The prevalence of all types of tinnitus was higher in women than in men, and it increased with age^1^. The contributing factors and/or etiologies of tinnitus were numerous, including hearing impairment, cardiometabolic disorders, sleep disturbances and sleep apnea, migraine, and non-migraine headache, etc.^2–4^. And, tinnitus often comorbided with other diseases, such as anxiety, depression, brain tumors or stroke, and some symptoms including irritability, inability to concentration, and sleep disturbances^5–7^.

Dysfunction in the cochlea, and neural reorganization in the auditory and non-auditory cortices have been reported to be associated with auditory tinnitus^8^. In addition to neuroinflammation and/or oxidative stress damages^9–13^, increased expression of N-methyl D-aspartate (NMDA) receptor (NR) gene^14^, decreasedγ-amino butyric acid (GABA) receptor (GR) and cannabinoid receptor genes were found in the cochlea and some brain areas^9,15^. Increased expresssion of K^+^–Cl^−^ cotransporter (KCC2) gene was also found in salicylate-induced tinnitus^16^. Besides, dysfunction of dopaminergic system was reported to be related with tinnitus formation^17,18^. On the other hand, the pathophysiology of periodic limb movements during sleep (PLMS) was reported to be related with dysfunction of dopaminergic system and/or calcium channels. And, dopamine agonists and α2δ calcium channel ligands are considered first-line treatments for PLMS and other sleep-related movement disorders^19^. Thus, it was reasonable to hypothesize that PLMS might be associated with tinnitus.

Currently, polysomnography is the gold standard and only clinically acceptable means of quantifying PLMS, which presented with repetitive stereotyped movements, typically in the lower limbs, during sleep. PLMS could disrupt sleep and result in daytime sleepiness^20^. PLMS have been proposed to be associated with increased risk of heart diseases and/or cardiovascular events^21^. Meta-analysis showed that, compared to subjects without PLMS, there were significantly higher co-morbidity rates of coronary artery disease and cardiovascular diseases (CVD), but not acute myocardial infarction, in subjects with PLMS^22^. Meanwhile, these PLMS-related complications and/or sequelae might also contribute to tinnitus formation^2,3,5–7^.

To our knowledge, the association of PLMS and tinnitus was never reported till now. Therefore, the aim of this study was to examine the association of PLMS and chronic tinnitus in humans.

## Methods

From Nov. 2011 to Jun 2017, clinical and overnight polysomnographic data of 2849 adult patients with sleep disturbances at Dalin Tzu Chi Hospital were retrospectively collected. The study was conducted in accordance with the Declaration of Helsinki and was approved by the Research Ethics Committee of Dalin Tzu Chi Hospital, Buddhist Tzu Chi Medical Foundation (No. B10604018). Informed written consent was waived because the study was a retrospective data analysis.

Clinical data including age, sex, subjective hearing loss, insomnia, and Parkinson’s disease were acquired before overnight PSG examination. Subjective hearing loss was graded as “no” and “yes” in response to a question of “Do you have difficulty hearing other people clearly in daily activities”. Insomnia was graded into five grades as “no”, “rare”, “sometimes”, “often”, and “usually”. PLMS was treated by two methods. First, PLMS was originally a continuous variable (/h). Second, PLMS was graded into four grades as “no”, “mild (>=5/h but <25/h)”, “moderate (>=25/h but <50/h), and “severe (>=50/h)”. And, chronic tinnitus was graded as “no” and “yes”, and regarded as non-tinnitus group and tinnitus group, respectively.

### Statistical analysis

Student’s t-test was used to test the difference of continuous variables between non-tinnitus and tinnitus group. Pearson’s Chi-Square test was used to test the association of PLMS with different severity and chronic tinnitus. Multivariate logistic regression was used to test the association of PLMS (continuous values) and chronic tinnitus. All analyses were performed using STATA 10.0 software (Stata Corp, College Station, Texas). P < 0.05 was considered to be significant.

## Results

There were 989 females and 1860 males in this study. The mean age was 50.6 years old (standard deviation, SD = 13.3, range=18~91) for all subjects. Among that, there were 1886 subjects without chronic tinnitus and 963 subjects with chronic tinnitus in this study.

Table 1 showed the basic characteristics of non-tinnitus and tinnitus groups. Compared to non-tinnitus group, tinnitus group had significantly higher age (mean =54.2 years old, SD = 12.2 versus mean=48.8 years old, SD = 13.5, p < 0.0001), proportion of females (39.4% versus 32.3%, p < 0.001), and prevalence of subjective hearing loss (37.7% versus 5.6%, p < 0.001) and insomnia (87.8% versus 76.5%, p < 0.001). But, PLMS (mean =1.1/h, SD = 3.4 versus mean=1.0/h, SD = 3.5, p = 0.4557) and prevalence of Parkinson’s disease (0.9% versus 0.8%, p = 0.808) were not significantly different between non-tinnitus and tinnitus groups.

**Table 1:** The characteristics of non-tinnitus and tinnitus groups.

	Non-tinnitus (n = 1886)	Tinnitus (n = 963)	p
Age (y/o)	48.8 (13.5)	54.2 (12.2)	<0.0001
Sex (F/M, %)	32.3/67.7	39.4/60.6	<0.001
Paroxysmal limb movement (/h)	1.0 (3.5)	1.1 (3.4)	0.4557
Subjective hearing loss (No/Yes, %)	94.4/5.6	62.3/37.7	<0.001
Parkinson’s disease (No/Yes, %)	99.2/0.8	99.1/0.9	0.808
Insomnia (grade, %) No Rare Sometimes Often Usually	23.5 25.7 25.4 16.9 8.5	12.2 14.2 25.8 31.2 16.6	<0.001

Table 2 showed the association of PLMS with different severity and chronic tinnitus. The severity of PLMS was not significantly associated with chronic tinnitus (p = 0.639 by Pearson’s Chi-Square test).

**Table 2:** The association between PLMS severity and chronic tinnitus.

	Paroxysmal limb movement (grade, %)				
	No (<5/h)	Mild (>=5/h but <25/h)	Moderate (>=25/h but <50/h)	Severe (>=50/h)	P
Non-tinnitus (n = 1886)	93.4	6.2	0.3	0.05	0.639
Tinnitus (n = 963)	92.4	7.3	0.3	0.00	

Table 3 showed the multivariate logistic regression analysis for the association of PLMS on chronic tinnitus with adjustment of other variables. The results showed that PLMS was not significantly associated with chronic tinnitus after adjusting for age, sex, subjective hearing loss, Parkinson’s disease, and insomnia.

**Table 3:** Multivariate logistic regression for tinnitus.

Variables	Coefficient	Standard error	P value
PLMS	0.983	0.013	0.207
Age	1.017	0.004	<0.001
Sex	0.775	0.075	0.008
Subjective hearing loss	9.761	1.254	<0.001
Parkinson’s disease	0.463	0.224	0.112
Insomnia	1.418	0.053	<0.001

*PLMS was treated as a continuous variable, and others were treated as category variables.

## Discussion

This retrospective study based on clinical and PSG data showed that PLMS, one kind of sleep disturbances, was not significantly associated with chronic tinnitus before and after adjusting other variables. This clinical finding did not support the expectation based on the dopaminergic system dysfunction for both of PLMS and chronic tinnitus^15,18,19^. Also, although PLMS-related complications and/or sequelae might contribute to tinnitus^2,3,5–7^, current results did not show positive correlation between PLMS and chronic tinnitus in humans at all.

In this study, we showed that older age, male subjects, hearing loss, and insomnia had higher risk for having chronic tinnitus. These findings were very reasonable and expectative. Talking about the relationship of tinnitus and sleep disturbance, loud tinnitus was associated with 2.8-fold and 3.3-fold increases in the odds ratios of insomnia in men and women, respectively^23^. Difficulty in initiating and maintaining sleep were related with tinnitus^23^. Furthermore, our previous studies showed that sleep disturbance, especially for sleep apnea, could increase the risk of tinnitus^2^. Thus, many people got trapped in a vicious cycle of sleep disturbances and tinnitus. Even though, it is still unclear whether sleep-related movement disorders, such as PLMS, was associated with tinnitus or not till now.

In the aspect of dopaminergic systemTrotti (2017), reported that dopamine agonists could reduce the severity of PLMS^19^. On the other hand, the results about tinnitus-related dopaminergic pathway dysfunction varied. For example, sulpiride, an agonists and antagonists of dopamine receptors (DRs) can reduce tinnitus^18^. Pramipexole, an agonist of DR2/3, could reduce tinnitus associated with presbycusis^24^. Bupropion, a dopamine reuptake inhibitor, did not enhance the treatment effect of repetitive transcranial magnetic stimulation of the temporal cortex for tinnitus^25^. Salicylate-induced tinnitus might be associated with increasing mRNA expressions of DR1A gene in the cochlear, brainstem and inferior colliculus, hippocampus and parahippocampus, and temporal lobes, but not in the frontal lobes^15^. So, it seemed that DR2/3 activation would suppress tinnitus, whereas DR1 activation would enhance tinnitus.

As for sleep-related movement disorders, although restless legs syndrome (RLS), PLMS, and periodic limb movement disorder (PLMD) were different by definition^26^, their treatments were very similar^27^. RLS might be associated with several conditions, including growing pains, kidney diseases, migraine, diabetes mellitus, epilepsy, rheumatologic disorders, CVD, liver and gastrointestinal disorders, and neuropsychiatric disorders [e.g., attention deficit hyperactivity disorder (ADHD), depression, and conduct disorder^28^. The prevalence of associated diagnoses of PLMS in pediatric population was 46.8% for OSA, 13% for ADHA, 9.1% for migraine, 9.1% for seizure, 6.5% for autism spectrum disorders, 9.1% for narcolepsy, and 96.6% for decreased serum ferritin^29^.

RLS and/or PLMS might also increase cardiovascular risk by increasing heart rate and blood pressure, resulting in sleep fragmentation and sleep deprivation, which produced adverse consequences for neural, metabolic, oxidative, inflammatory, and vascular systems, and iron deficiency, which was also a risk for CVD^30^. Also, PLMS are found in 30% of patients suffering from OSA^31^. Patients with PLMS had higher artery stiffness than their controls and those with moderate/severe OSA did. And, a possible additive effect of OSA and PLMS on arterial stiffness was found^32^. Thus, PLMS might increase the risk of CVD directly and/or indirectly via its comorbid OSA. However, we found that PLMS was not related with tinnitus. Otherwise, it was possible that PLMS might be related with tinnitus indirectly via its comorbid diseases, including OSA as our previous study shown^2^.

This finding did not against the use of DR agonists and/or antagonists on chronic tinnitus clinically. We still supposed that some DR agonists might be helpful for patients with PLMS and/or chronic tinnitus by reducing the chances of PLMS-related sequelae. But, it needs more studies to confirm our hypothesis in the future.

## Conclusion

This clinical study found that PLMS was not associated with chronic tinnitus in humans, even though possible similar pathopysiology was established in both symptoms.
